# Distinct efficacy of HIV-1 entry inhibitors to prevent cell-to-cell transfer of R5 and X4 viruses across a human placental trophoblast barrier in a reconstitution model *in vitro*

**DOI:** 10.1186/1742-4690-5-31

**Published:** 2008-03-31

**Authors:** Ahidjo Ayouba, Claude Cannou, Marie-Thérèse Nugeyre, Françoise Barré-Sinoussi, Elisabeth Menu

**Affiliations:** 1 Institut Pasteur, Département de Virologie, Unité Régulation des Infections Rétrovirales, 25 rue du Dr Roux, 75724 Paris cedex 15, France

## Abstract

**Background and methods:**

HIV-1 cell-to-cell transmission is more efficient than infection of permissive cells with cell-free particles. The potency of HIV-1 entry inhibitors to inhibit such transmission is not well known. Herein, we evaluated the efficacy of this new class of antiretrovirals to block cell-to-cell transmission of HIV-1 in a model of reconstitution of the human placental trophoblast barrier *in vitro*.

**Results:**

Our data show that CCR5 antagonists and T20 inhibit the passage of the virus across the BeWo cell monolayer in contact with PBMCs infected with an R5 (Ba-L) and a dualtropic (A204) HIV-1 with IC50s in the range of 100 – 5,000 nM for TAK779; 90 to 15,000 nM for SCH-350581 and 3,000 to 20,000 nM for T20. The CXCR4 antagonist AMD3100 is also effective against X4 HIV-1 infected PBMCs in our model with IC50 comprised between 4 nM and 640 nM. HIV-1 entry inhibitors are less efficient to block cell-to-cell virus transmission than cell-free HIV-1 infection of PBMCs and CCR5 antagonists do not prevent PBMC infection by dual tropic HIV-1 in contrast to cell-to-cell infection in our model.

Surprisingly, T20 (and C34) do not block cell-to-cell transmission of X4 HIV-1 but, rather, increase 80 to 140 fold, compared to control without drug, the passage of the virus across the trophoblast barrier. Additional experiments suggest that the effect of T20 on BeWo/PBMC-X4 HIV-1 is due to an increase of effector-target cells fusion.

**Conclusion:**

Our results support further evaluation of HIV-1 coreceptor antagonists, alone or combined to other antiretrovirals, in a perspective of prevention but warn on the use of T20 in patients bearing X4 HIV-1 at risk of transmission.

## Background

Human Immunodeficiency Virus Type-1 (HIV-1) transmission occurs by direct contact of cell-free virions and permissive cells, by interaction between infected and uninfected target cells (*cis *infection), or by viral transfer in *trans *between an uninfected cell (like Dendritic cells or B cells) and CD4 positive T cells through virological synapses [1-3]. For both sexual and mother-to-foetus transmissions, infection in *cis *via cell-to-cell contact was shown to be more efficient than infection with cell-free virus [4]. Hence, sexual and mother-to-foetus transmission occurred, although at lower rates, despite plasma viral load suppression [5,6]. This observation highlights the difficulty to fully block HIV-1 cell-to-cell infection and transmission, even in the presence of neutralizing antibodies or antiretrovirals [7,8].

In the particular case of HIV-1 mother-to-child transmission (MTCT), remarkable progress has been made since the use of antiretroviral (ARVs) drugs [9] for prevention, together with safer obstetrical interventions and babies formula feeding. Thus, in Europe and North America, HIV-1 MTCT has been lowered to less than 2% [10-12], now occurring mainly *in utero *[13]. In lower income countries as in Africa and Asia, HIV-1 MTCT remains a public health issue as more than 90% of the 2,000 children infected daily originated from these parts of the world [14]. Most of these were infected via the mother-to-child route. This situation is hopefully improving, thanks to the introduction of ARVs regimen proven efficacious in clinical trial settings or pilot programmes [15-17].

All ARVs currently used for HIV-1 positive patients care or for prevention of MTCT (PMTCT) are either nucleosidic analogs, non-nucleosidic inhibitors of HIV-1 reverse transcriptase or Protease inhibitors. Some of these drugs, like AZT, have been in use since the beginnings of AIDS therapy twenty years ago. Because of their long term usage, ARV lead to selection of drug resistant HIV, thus diminishing their efficacy [18]. A fourth category of ARVs has emerged as HIV-1 entry inhibitors, consisting of coreceptors antagonists and fusion inhibitors. T20 (Enfuvirtide^®^) is the prototype of fusion inhibitor that is now in routine clinical practice for a category of patients who had experienced all the available drugs and whose viruses had become resistant to [19]. The other type of entry inhibitors (CCR5 and CXCR4 antagonists principally) is under intensive evaluation [20-22]. Some of them have been evaluated successfully as microbicides against vaginal, cell-free SHIV, in macaques, conferring a full protection when used in combination and at high doses, in the millimolar range [23,24]. The potential toxicity of these high doses of drugs, upon long term use or repeated usage, is however, yet unknown. Other drugs like Maraviroc have undergone successful phase IIII clinical trials [25] and are now cleared by the US FDA and the European Union for patient's care.

Primary human term placental trophoblast cells and the placental derived choriocarcinoma cell line BeWo express very little or not at all surface CD4 molecules, according to different studies [26,27]. Both cell types naturally express the two major HIV-1 coreceptors, CCR5 and CXCR4 on their surfaces [28-31]. Attempts to infect placental trophoblasts or BeWo cells with cell-free viruses lead to resistance or unproductive infection, by mechanisms still debated [31,32]. In contrast, placental trophoblast cells are permissive to cell-to-cell infection [4,33]. We, and others, use single cycle replication VSV-G pseudotyped HIV-1 to allow viral entry for cell-free virus infection of BeWo cells [31] or placental explants [34]. Alternatively, our group has developed an in vitro model of the polarized human trophoblast barrier to study the mother/fetus interface *in vitro *in direct contact with HIV-1 infected PBMCs [4,33] that we used in the present study.

To anticipate the future by evaluating therapeutic and/or preventive strategies complementary to those existing, we used in the present study this *in vitro *model of the human placental trophoblast barrier to evaluate the potency of CCR5 inhibitors (TAK779 and SCH-350581), CXCR4 antagonist (AMD3100) and fusion inhibitors (T20 and C34) to prevent the passage of HIV-1 across a monolayer of BeWo cells by virus infected PBMCs. Our data show that CCR5 and fusion inhibitors, in one hand, and the CXCR4 antagonist AMD3100, in the other hand, efficiently blocked the passage of R5 or R5X4 and X4 HIV-1, respectively, through the BeWo monolayer. However, HIV-1 entry inhibitors are less efficient to block cell-to-cell virus transmission than cell-free HIV-1 infection of PBMCs and CCR5 antagonists do not prevent PBMC infection by dual tropic HIV-1 in contrast to cell-to-cell infection in our model.

Surprisingly, T20 and C34 could not inhibit the passage of the virus across the BeWo cell monolayer by PBMC infected with X4 HIV-1. Rather, they increased viral passage and thus suggest that T20, the unique entry inhibitor approved for therapy so far, is not efficient to block X4-HIV-1 transmission in this context of cell-to-cell contact and raise the question of its potential use in HIV-1 PMTCT interventions.

## Results

### Lower efficacy of T20, TAK779, SCH-350581 and AMD3100 to block cell-to-cell virus transmission than cell-free HIV-1 infection

To evaluate the efficacy of the different HIV-1 entry inhibitors in blocking the passage of the virus across a human placental trophoblast barrier *in vitro*, we compared the 50% inhibitory concentrations (IC50s), of T20, TAK779, SCH-350581 and AMD3100 determined in the classical cell-free virus/PBMCs infectivity assay to those in our *in vitro *model of the placental barrier using HIV-1 infected PBMCs in contact with BeWo cells.

**Figure 1:**
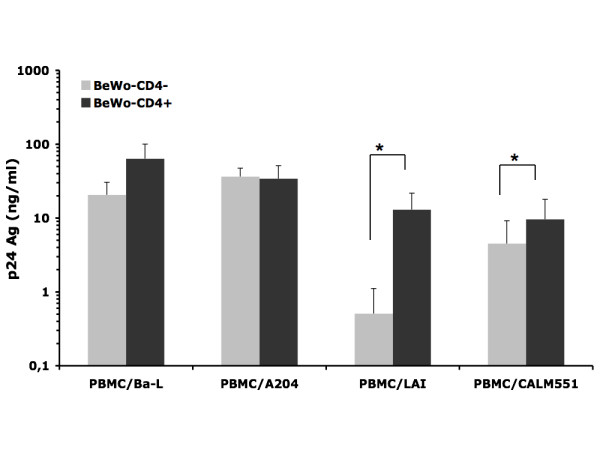
**Basal HIV-1 p24 antigen production by indicator PBMCs in the basolateral chamber of the in vitro trophoblast barrier model**. After 3 h contact between PBMCs infected with the indicated viral isolates (effector cells) and BeWo cells (targets), the Transwell^® ^inserts were extensively washed and left in culture in plates containing activated, HIV-1 negative, indicator PBMCs for 5 days. Inserts were then removed, and cultures of indicator PBMCs were continued for 3 more days and HIV-1 p24 core antigen was assayed in the supernatants. The figure shows the average (mean ± SD) of at least 2 independent experiments performed in triplicates. The grey bars represent target CD4 negative BeWo cells and the black bars represent target CD4 positive BeWo cells. The asterix on the top of the bars denotes a statistically significant difference (p = 0.02) by Mann-Whitney U test.

All the experiments with BeWo cells were performed with the cells expressing CD4 or not, unless otherwise mentioned. After 3 hours of contact between BeWo cells and PBMCs infected with HIV-1 of different phenotypes, indicator PBMCs in the basolateral compartment produced detectable amounts of p24 antigen, 8 days post-contact (Figure 1). Large quantities (up to 100 ng/ml) of p24 antigen were detected when R5 or R5X4 virus infected PBMCs were used for the contact. Lower quantities were detected when using PBMCs infected with the X4 HIV-1 isolates LAI and KH025. The contact between infected PBMCs and BeWo cells led to the passage of HIV-1 across the placental trophoblast barrier *in vitro*, independently of the expression of CD4 on BeWo cells surface. Although the amounts of HIV-1 p24 antigen were found generally higher in basolateral chambers when CD4 positive BeWo cells were used, the difference was only statistically significant when effector PBMCs were infected with HIV-1-LAI and KH025 (p = 0.01 and 0.04 respectively, Mann-Whitney U test).

**Figure 2:**
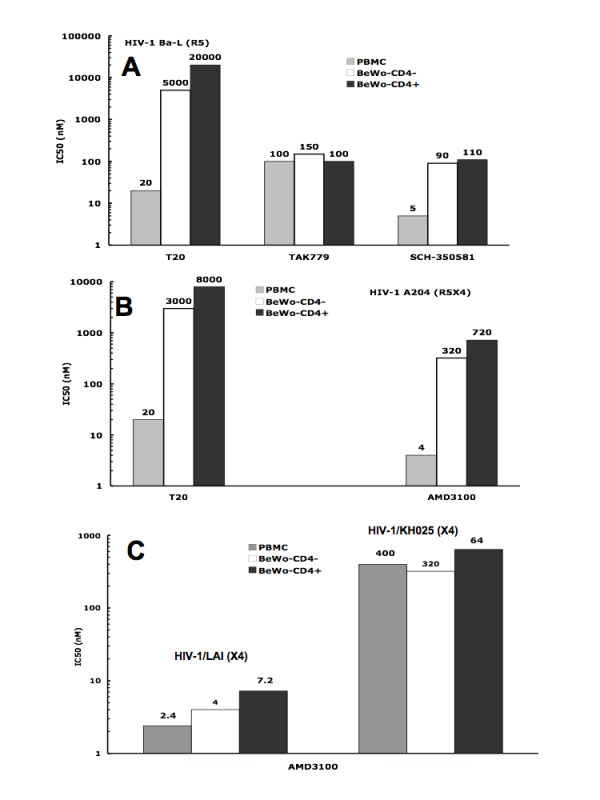
**Distinct efficacy of entry inhibitors to block cell-free HIV-1 infection and cell-to-cell virus transmission**. Activated human PBMCs (10^5^) were incubated for 30 min with the different entry inhibitors at various concentrations and then infected with HIV-1 Ba-L (R5), A204 (R5X4), LAI (X4) or KH025 (X4) for 1 h at 37°C in the presence of drugs at varying doses. After 3 washes, cells were resuspended in RPMI1640 containing IL-2 and the same drug they were pre-incubated with. HIV-1 infected PBMCs were then left in culture for 3 days, when the culture supernatants were replaced with RPMI1640-IL-2 medium without drug. The cultures were continued for 4 more days and HIV-1 p24 antigen was assayed in the supernatants. For the inhibition of HIV-1 passage by entry inhibitors, BeWo cells (CD4 negative, white bars or CD4 positive, black bars) were pre-incubated for 30 min at 37°C in the presence of varying doses of TAK779, SCH-350581, or AMD3100. If T20 or C34 were used as inhibitors, PBMCs infected with different HIV-1 isolates were pre-incubated with varying doses of T20 or C34 for 30 minutes before the contact with BeWo cells. At this point, HIV-1 Ba-L, HIV-1 A204, HIV-1 LAI or HIV-1 KH025 infected PBMCs were added and both cell types were left in contact for 3 h at 37°C. Extensive washes were then performed and the Transwell^® ^inserts transferred on new plates containing activated HIV negative indicator PBMCs, without drugs. The inserts were removed 5 days later and the culture continued 3 more days, when the HIV-1 p24 core antigen was quantified in the supernatants. The IC50s were determined graphically from the titration curves. Figure 2A: comparison of IC50s during the inhibition of cell-free HIV-1 Ba-L infection of PBMC (grey bars), HIV-1 Ba-L/CD4 negative BeWo cells (white bars) and HIV-1 Ba-L PBMC/CD4 positive BeWo cells (black bars). Figure 2B: the PBMCs were infected with HIV-1 A204. Figure 2C: the PBMCs were infected with HIV-1 LAI or HIV-1 KH025 and the inhibition was achieved by AMD3100.

We next compared the IC50s of T20, TAK779 and SCH-350581 in infectivity assay with cell-free HIV-1-Ba-L (R5) and in the two chamber system using PBMCs infected with the same isolate and BeWo cells. Results from Figure 2A show that of the three drugs tested, SCH-350581 is the most potent inhibitor of the infection by cell-free HIV-1/Ba-L with an IC50 of 5 nM, followed by T20 at 20 nM and finally, TAK779 with an IC50 of 100 nM. Comparison of IC50s determined in the PBMC infectivity assay and those observed in the double chamber system shows for HIV-1/Ba-L an increase for the three drugs tested. Thus, for T20, this increase was 250 and 1,000 folds for CD4 negative and positive BeWo cells, respectively. For TAK779, a moderate 1.5 fold increase of IC50 was observed for CD4 negative BeWo cells, and equivalent potency for CD4 positive BeWo cells. For SCH-350581, 90 and 110 nM of the drug was necessary to achieve 50% inhibition of HIV-1 passage across the monolayer of CD4 negative and positive BeWo cells, respectively. These concentrations are roughly 20 times those observed for the inhibition of PBMC infection with cell-free HIV-1-Ba-L.

Next, we compared the IC50s of T20, TAK779, SCH-350581, and AMD3100 during infection of PBMCs with the dualtropic R5X4 HIV-1-A204 and during the contact between PBMCs infected with the same isolate and BeWo cells (Figure 2B). Only the fusion inhibitor T20 and the CXCR4 antagonist AMD3100 were able to inhibit PMBC infection by this dualtropic HIV-1 isolate. The IC50s were 20 and 4 nM for T20 and AMD3100, respectively. The CCR5 antagonists TAK779 and SCH-350581 could not efficiently and in a dose dependent manners inhibit the infection of PBMCs by this R5X4 HIV-1 isolate. TAK779 inhibited 80% of viral replication at 20 nM followed by an increase up to the level without drug at 2,000 nM of the drug. For SCH-350581, the pattern was different as a constant 20% inhibition of viral replication was observed in the range 10 to 2,000 nM of drug. Failure of CCR5 antagonists to prevent PBMC infection by dualtropic HIV-1 has been reported recently [35]. During the interaction between PBMCs infected with the A204 HIV-1 isolate and BeWo cells, all the four drugs potently inhibited viral passage from the apical to the basolateral sides of the placenta trophoblast barrier. Here again, the concentrations necessary to achieve 50% inhibition of viral passage were higher when compared to those observed in cell-free HIV-1-A204 infection of PBMCs. Hence, for T20 the IC50s were 3,000 and 8,000 nM for BeWo cells CD4 negative and positive, respectively. These concentrations were 150 and 400 times higher than those we observed for the inhibition of cell-free A204 infection of PBMCs. For the CXCR4 antagonist AMD3100, the concentrations needed for 50% inhibition of HIV-1 A204 passage across the BeWo cell monolayer were 320 and 720 nM, for the CD4 negative and positive cell lines, respectively (Figure 2B). On the contrary to what was observed during cell-free HIV-1-A204 infection of PBMCs, the CCR5 antagonists TAK779 and SCH-350581 inhibited viral passage upon cell-to-cell contact, though at high concentrations. Thus, for TAK779, the IC50s were 5,000 and 100 nM during PBMC/HIV-1 A204 interaction with BeWo CD4 negative and positive, respectively. For SCH-350581, the 50% inhibitory concentrations were even higher: 5,000 and 15,000 nM for BeWo CD4 negative and positive, respectively, in contact with A204 infected PBMCs.

Finally, we compared the IC50s of AMD3100 during the infectivity assay of PBMCs with cell-free X4 HIV-1-LAI and HIV-1-KH025 and during the contact between PBMCs infected with the same isolates and BeWo cells. Results from Figure 2C indicated that AMD3100 inhibited with different efficacies PBMC infection by the two X4 HIV-1 isolates. The IC50s observed were 2.4 nM and 400 nM for HIV-1-LAI and HIV-1-KH025, respectively. The concentrations of AMD3100 necessary to achieve 50% inhibition of HIV-1-LAI passage across the BeWo cells monolayer were 1.6 and 3 times, for CD4 negative and positive BeWo cells, respectively, higher than the IC50 required to block cell-free virus infection of PBMC. Much higher concentrations of AMD3100 were necessary to reach the 50% inhibitory concentrations that block HIV-1 KH025 passage across the BeWo monolayer. IC50s of 320 nM and 640 nM were observed during the interaction between PBMC infected with KH025 and CD4 negative and positive BeWo cells, respectively.

Altogether, these data indicate that HIV-1 entry inhibitors are less efficient, *in vitro*, to block cell-to-cell virus transmission than cell-free HIV-1 infection of PBMCs and that CCR5 antagonists do not prevent PBMC infection by dual tropic HIV-1 in contrast to cell-to-cell infection in our model.

### The fusion inhibitors T20 and C34 increase the viral passage across the BeWo cell line monolayer in contact with PBMCs infected with X4 HIV-1 isolates

**Figure 3:**
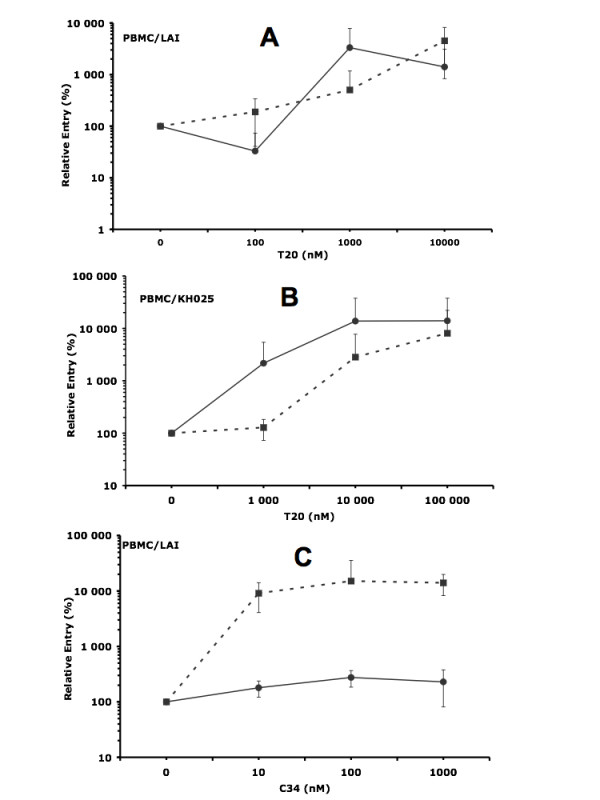
**The fusion inhibitors T20 and C34 enhance viral passage across the BeWo cell monolayer in contact with PBMCs infected with X4 HIV-1**. PBMCs infected with HIV-1 LAI or HIV-1 KH025 isolates were pre-incubated with varying doses of T20 or C34 for 30 minutes. Then, HIV-1 LAI or HIV-1 KH025 infected PBMCs were added to the BeWo cell monolayer and were left in contact for 3 h at 37°C. Extensive washes were then performed and the Transwell^® ^inserts transferred on new plates containing activated HIV negative indicator PBMCs, without drugs. The inserts were removed 5 days later and the culture continued 3 more days, when the HIV-1 p24 core antigen was quantified in the supernatants. The figures show HIV-1 passage across the model of the trophoblast barrier normalized to the control (without drugs) as a function of T20 (or C34) concentration. Continuous line, CD4 positive BeWo cells as target cells; dashed line: CD4 negative BeWo cells as target cells. **3A**: PBMCs infected with HIV-1 LAI as effector cells in the presence of T20; **3B**: PBMCs infected with HIV-1 KH025 as effector cells in the presence of T20 and **3C**: PBMCs infected with HIV-1 LAI as effector cells in the presence of C34.

The fusion inhibitor T20, although efficient in blocking PBMC infection by cell-free HIV-1-LAI and HIV-1-KH025 with IC50s of 20 and 5 nM respectively, was unable to inhibit the passage of the virus across the BeWo cells monolayer in contact with PBMCs infected with these X4 viruses. On the contrary, we found that T20 increases by 80 to 140 folds the level of passage without drug of both viruses across the monolayer of BeWo cells, in a dose-dependent manner as shown on Figure 3A &3B. This enhancement started at concentrations of 1,000 nM of drug and was independent of the presence of CD4 on the surface of BeWo cells. At 100 nM of T20, 60% of inhibition of viral passage was observed after the interaction between HIV-1/LAI infected PBMCs and CD4 positive BeWo cells (Figure 3A, continuous line), followed by an increase above the control (without T20) level at 1,000 nM of the drug. Results obtained with a different batch of PBMCs infected with HIV-1/LAI or HIV-1/KH025 (not shown) were consistent with those presented herein.

To confirm this result, we also used another fusion inhibitor, C34, a 34 amino-acid peptide from HIV-1 heptad repeat-2 (HR2), like T20, and whose mechanism of action is similar to T20, but that lacks 12 amino acids at the C-terminus end of T20. In addition, C34 does not bind cell membrane on the contrary of T20 [36]. Hence, if the enhancement we observed was due to BeWo cell membrane disorganisation by T20, C34 should not induce such increase of viral passage. Results obtained with C34 on cell-free virus (Ba-L and LAI) PBMC infection were consistent with data from the literature, with IC50s of 5 nM for Ba-L and 0.8 nM for LAI (data not shown). When C34 was used to block viral passage across the monolayer of BeWo cells in contact with PBMCs infected with HIV-1/LAI, an increase of viral passage was observed (Figure 3C). This increase of HIV-1/LAI passage across the BeWo monolayer was stronger when BeWo cells were CD4 negative (figure 3C, dashed line). In that case, an increase of more than 100 folds the control level was observed at 100 nM C34, in contrast to the 1.7–2.3 folds observed with BeWo CD4 positive cells (Figure 3C, continuous line).

### T20 increases fusion between BeWo cells and PBMC infected with HIV-1 LAI

**Figure 4:**
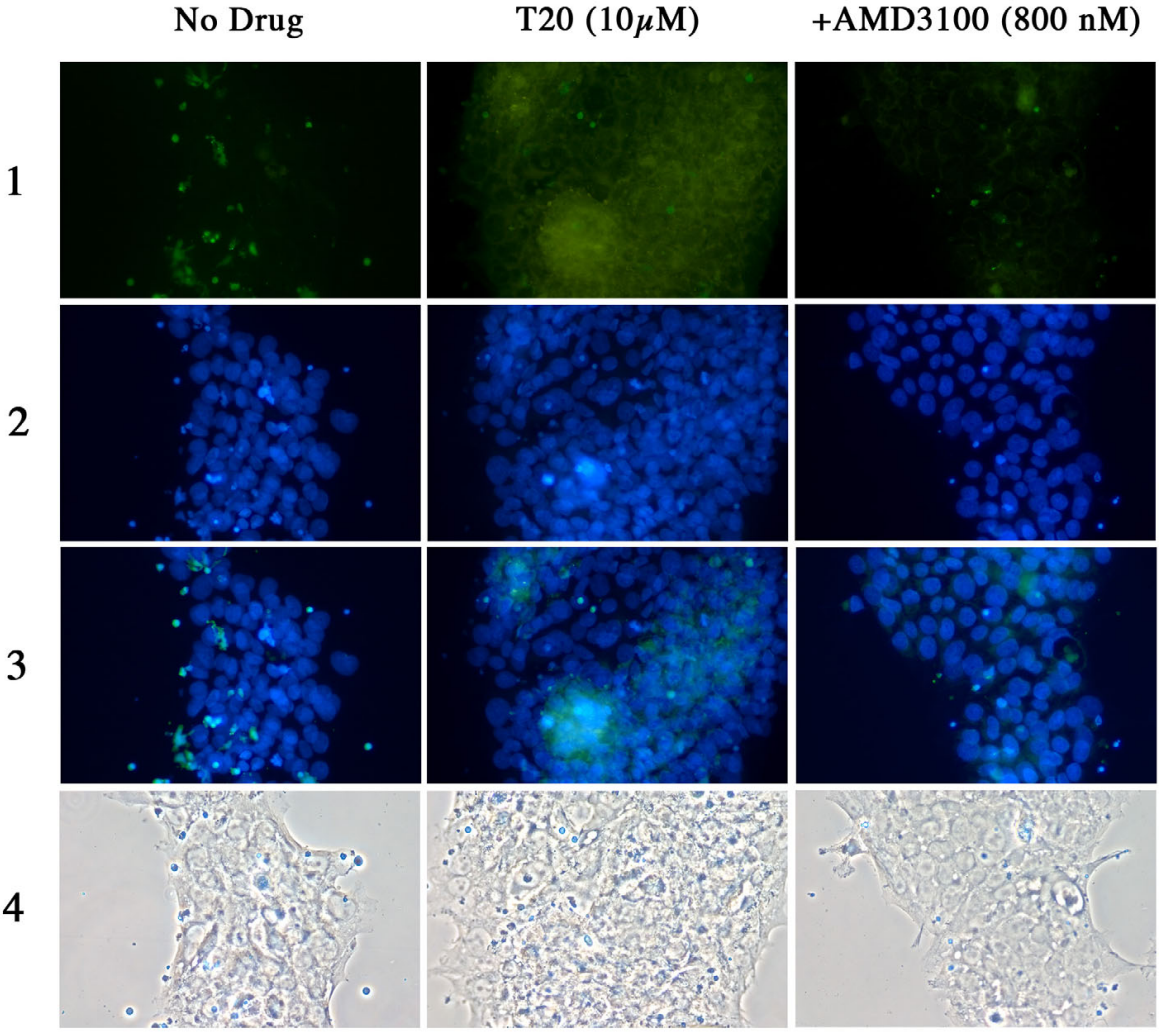
**The fusion inhibitor T20 significantly enhances target CD4 negative BeWo cells-HIV-1/LAI infected PBMCs fusion**. Target CD4 negative BeWo cells (2 × 10^4^/well) were coated overnight on slides as described in the method section. Target cells were then pre-incubated with indicated doses of AMD3100 or T20, or without drug, for 30 minutes at 37°C. At this point, calcein-AM loaded, HIV-1 LAI infected PBMCs were added ant left to interact for 5 h at 37°C. Slides were then washed and the cells fixed. The nuclear dye DAPI was then added into the wells for 5 min and washed out afterwards. The slides, once dried, were mounted in the presence of an antifade reagent at left to cure overnight at +4°C, in the dark. The day after, slides were observed under fluorescent microscope (20× magnification) and the images captured. Phase contrast images were also captured. The figure shows representative microscope fields of 2 (no drug or AMD3100) or 4 different wells (T20) for each experimental conditions tested. Row 1: Calcein stain; row 2: DAPI stain; row 3: merge of 1 and 2; row 4: phase contrast images. The green spots and green areas are intact PBMCs and their fused cytoplasm with BeWo cells, respectively. The blue spots are cell nuclei coloured by DAPI.

**Figure 5:**
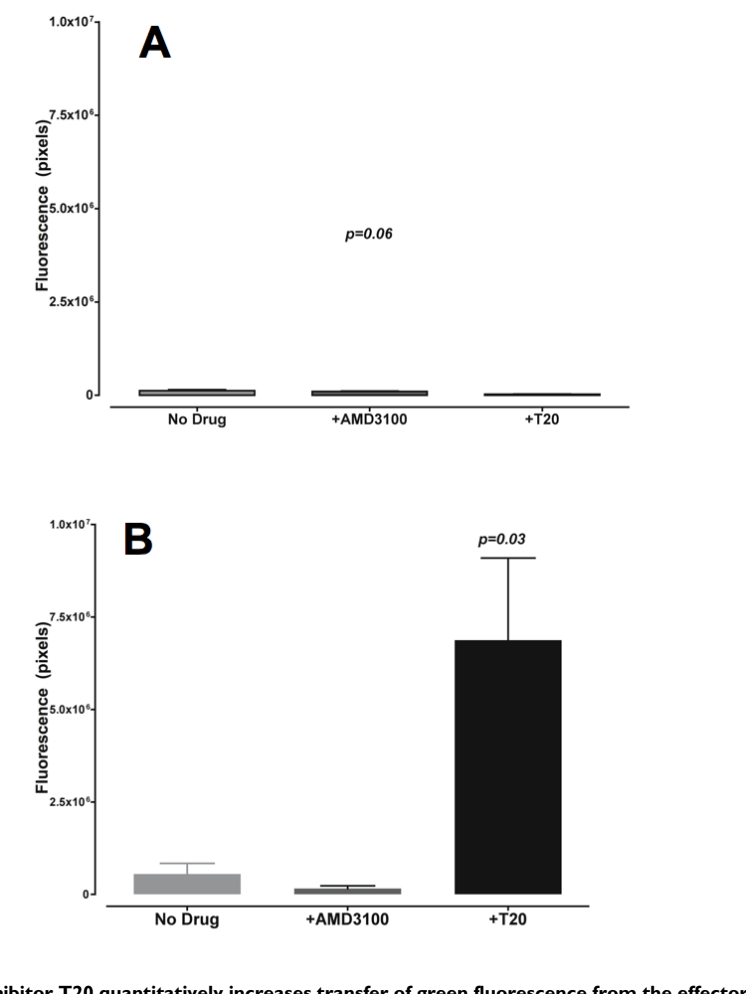
**The fusion inhibitor T20 quantitatively increases transfer of green fluorescence from the effector to target CD4 negative BeWo cell cytoplasms**. Green fluorescence from the experiment on slides (figure 4) was quantified in light units (pixels) inside and outside CD4 negative BeWo cells. Figure 5A (upper panel) represents the green fluorescence in PBMCs that have adhered on the surface of BeWo cells (outside). Figure 5B (lower panel) represents green fluorescence that has been transferred from HIV-1 LAI infected PBMCs into BeWo cells (inside). The figure shows averaged green fluorescence intensities from 3 differents fields in the absence of drug, in the presence of 10 μM T20 or 800 nM AMD3100. Histograms represent means and the error bars are standard deviation to the mean.

**Figure 6:**
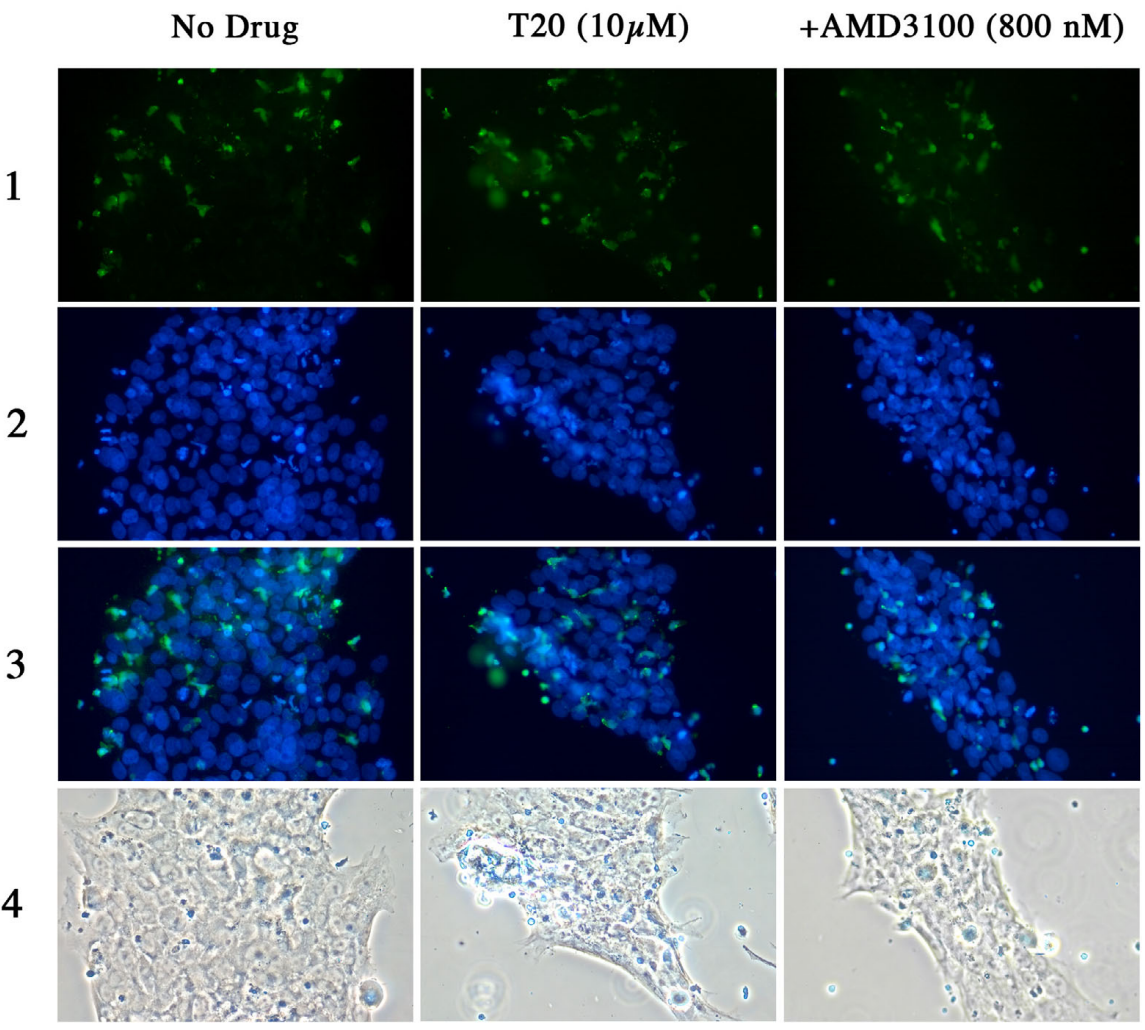
**The fusion inhibitor T20 moderately enhances target CD4 positive BeWo cells-HIV-1/LAI infected PBMCs fusion**. Target CD4 positive BeWo cells (2 × 10^4^/well) were coated overnight on slides as described in the method section. Target cells were then pre-incubated with indicated doses of AMD3100 or T20, or without drug, for 30 minutes at 37°C. At this point, calcein-AM loaded, HIV-1 LAI infected PBMCs were added ant left to interact for 5 h at 37°C. Slides were then washed and the cells fixed. The nuclear dye DAPI was then added into the wells for 5 min and washed out afterwards. The slides, once dried, were mounted in the presence of an antifade reagent at left to cure overnight at +4°C, in the dark. The day after, slides were observed under fluorescent microscope (20× magnification) and the images captured. Phase contrast images were also captured. The figure shows representative microscope fields of 2 (no drug or AMD3100) or 4 (T20) different wells for each experimental conditions tested. Row 1: Calcein stain; row 2: DAPI stain; row 3: merge of 1 and 2; row 4: phase contrast images. The green spots and green areas are intact PBMCs and their fused cytoplasm with BeWo cells, respectively. The blue spots are cell nuclei coloured by DAPI. The figure shows moderate enhancement of viral passage by T20 on CD4 positive BeWo cells.

**Figure 7:**
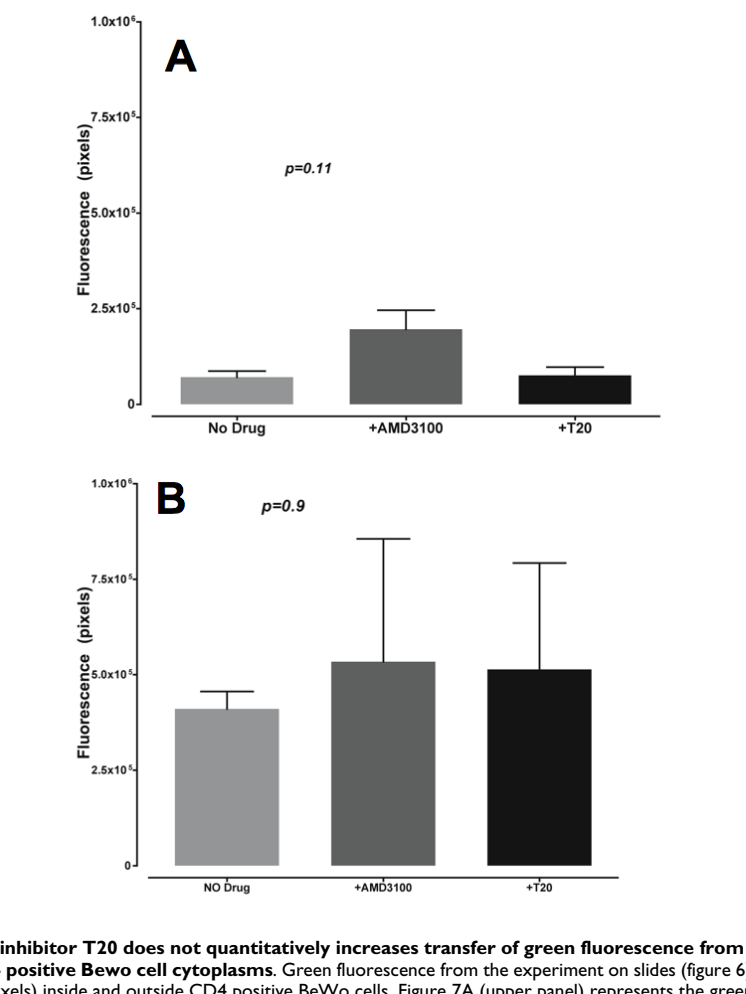
**The fusion inhibitor T20 does not quantitatively increases transfer of green fluorescence from the effector to target CD4 positive Bewo cell cytoplasms**. Green fluorescence from the experiment on slides (figure 6) was quantified in light units (pixels) inside and outside CD4 positive BeWo cells. Figure 7A (upper panel) represents the green fluorescence in PBMCs that have adhered on the surface of BeWo cells (outside). Figure 7B (lower panel) represents green fluorescence that has been transferred from HIV-1 LAI infected PBMCs into BeWo cells (inside). The figure shows averaged green fluorescence intensities from 3 differents fields in the absence of drug, in the presence of 10 μM T20 or 800 nM AMD3100. Histograms represent means and the error bars are standard deviation to the mean. There is no difference statistically significant between the three different conditions compared (no drug, +T20, +AMD3100).

To further investigate why the fusion inhibitor enhanced X4 HIV-1 passage across the BeWo cells monolayer, we checked if this increase was related to an increase of BeWo-PBMC cell membranes fusion. To that end, we performed dye transfer experiments using effector PMBCs infected with HIV-1 LAI loaded with the green cytoplasmic dye calcein-AM and BeWo cells as targets, seeded on slides. Dye transfer from the cytoplasm of the effector cell to that of the target cell evidenced the fusion between effector and target cells membranes. Figure 4**and **6 show representative examples of such experiments performed with CD4 negative and positive BeWo cells, respectively. The 3 columns represent the 3 different experimental conditions tested as indicated on the top of each of them. The middle column from figure 4 shows massive intrusion of green fluorescence into the cytoplasm of target CD4 negative BeWo cells in the presence of 10 μM T20. This green fluorescence inside BeWo cells corresponds to the transfer, ensuing cell membrane fusion, of the cytoplasm of HIV-1 infected effector PBMC into BeWo cells. The quantification of green fluorescence outside and inside Bewo cells from pictures of figure 4 is presented on figure 5A**and **5B**respectively**. T20 dramatically increased the quantity of green fluorescence (in pixels) transferred into the cytoplasm of CD4 negative BeWo cells (p = 0.03). The same experiments were performed in parallel with CD4 positive BeWo cells, as shown on figures 6 and 7. The difference in fusion/adhesion in the presence or not of T20 was not statistically significant, corroborating the observations from figure 3. As controls, slides were performed in the presence of 800 nM AMD3100 (**column on the right of **figures 4**and **6). AMD3100 blocked HIV-1 LAI infected PBMC outside CD4 positive BeWo cells (figure 7A). Experiments from figures 4, 5, 6 and 7 revealed that the enhancement of X4 virus passage by T20 we observed in the double chamber system was in fact due to an increase in cell fusion. The quantification (Figure 5 &7) in light units of green fluorescence outside BeWo cells (hence still inside infected PBMC) (Figure 5A**and **7A) and inside BeWo cells (Figure 5B**and **7B) is consistent with this scheme. Green fluorescence inside BeWo cells (Figure 5B) was dramatically significantly increased only in CD4 negative BeWo cells.

At this point, we concluded that T20, by increasing the fusion of PBMCs infected with HIV-1/LAI on BeWo cells, facilitated the entry of the virus into these target cells especially in wildtype BeWo cells which do not express CD4 at their surface.

### T20 efficiently blocks viral passage across a human placenta-derived cell line monolayer in contact with CD4+ T cells infected by the X4 HIV-1/LAI

PBMCs are constituted of various cell types acting on each other either by direct cell-to-cell interaction, or via soluble factors such as cytokines. In a further attempt to identify on which of these two alternatives T20 acts to increase X4 HIV-1 infected PBMCs passage across the BeWo cell monolayer, we purified CD4 positive T cells from PBMCs (from the same batch used in experiments above) and infected them with HIV-1 LAI. We then performed the contact with the BeWo cell monolayer in our *in vitro *model in the same conditions as with PBMCs.

**Figure 8:**
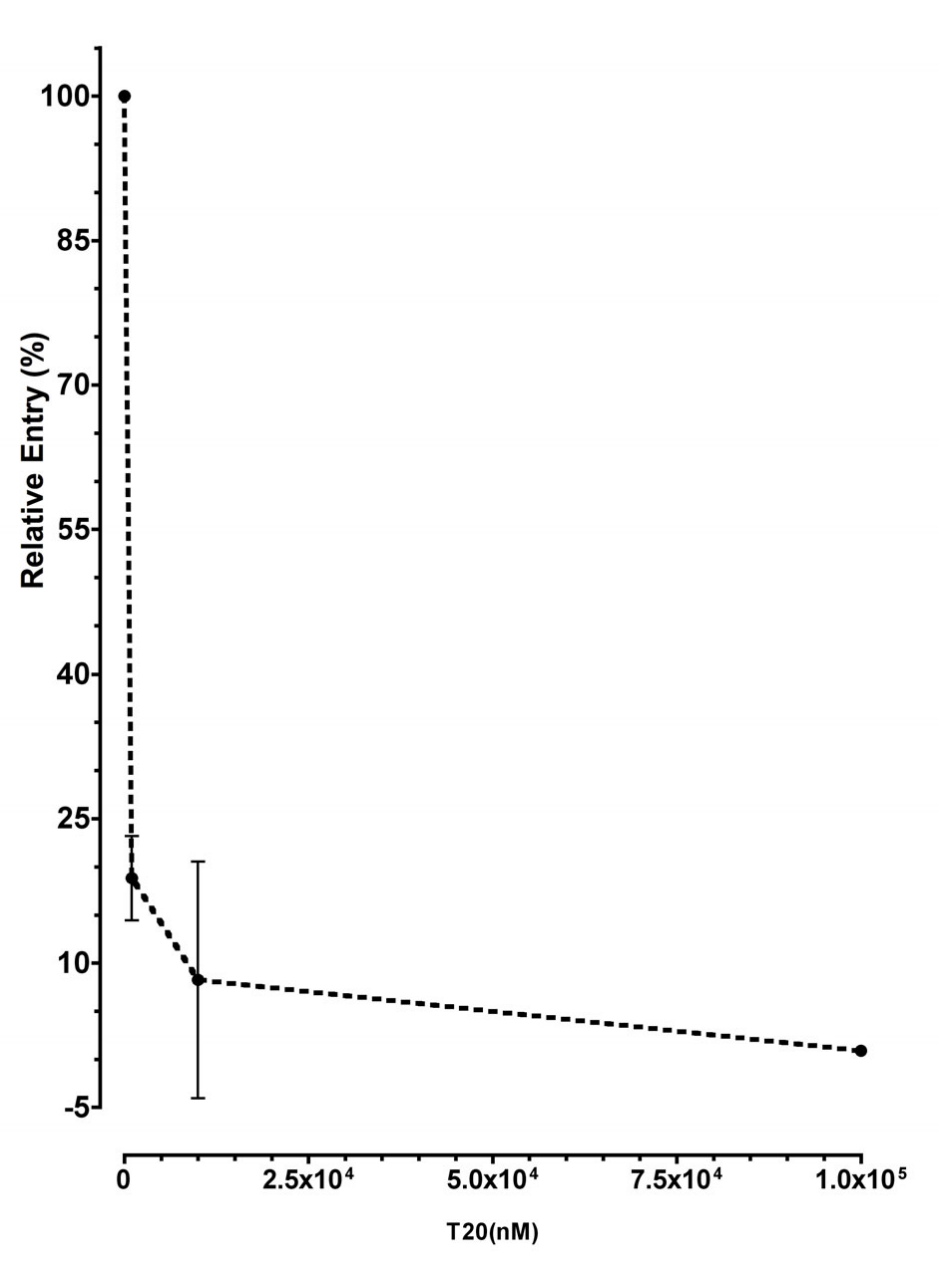
**The fusion inhibitor T20 inhibits viral passage across the BeWo monolayer in contact with HIV-1/LAI infected CD4+ T cells**. The experiment was designed as described for figure 3, except that purified CD4 positive T cells were used as effector cells, instead of whole PBMCs, for infection with HIV-1 LAI. The figure shows a dose-dependent inhibition of the interaction between BeWo-CD4 negative target cells and CD4+ T cells infected with HIV-1/LAI.

Results from this experiment showed that T20 was highly efficient to block viral passage when BeWo cells were in contact with purified CD4 positive T cells infected with the X4 HIV-1/LAI (figure 8). In this situation, the IC50 and IC90 observed were 1,200 nM and 2,000 nM, respectively. A complete blockade of viral passage was observed at T20 concentration below 5,000 nM (figure 8). From this experiment, we concluded that cell types other than CD4 positive T-cells present in PBMCs are responsible, either directly, or indirectly through soluble factors, of T20 increase of X4 viral passage across the BeWo cell monolayer.

### A soluble factor secreted by CD4 negative cells from PBMCs contributes to the increase of viral passage through the BeWo cells monolayer by T20

**Figure 9:**
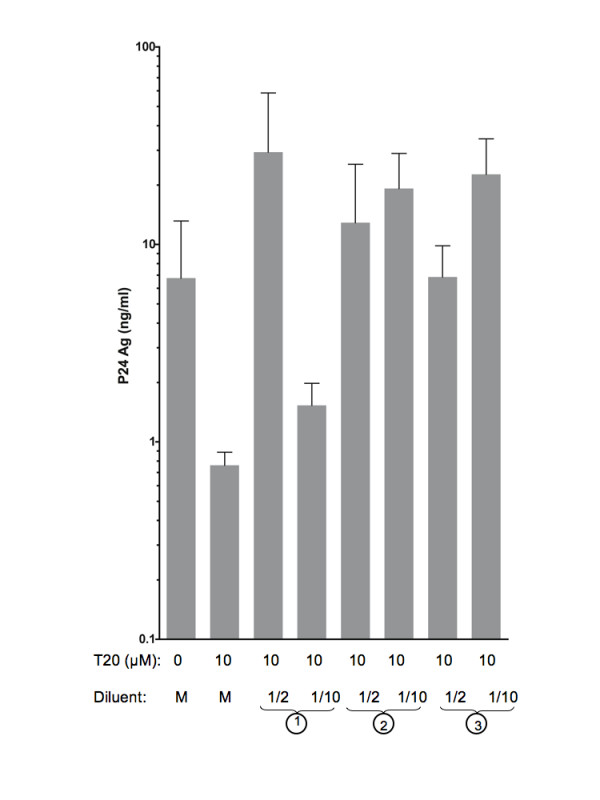
**A soluble factor secreted by CD4 negative cells from PBMCs contributes to the increase of viral passage through the BeWo cell monolayer by T20**. Purified CD4+ T-cells were infected with HIV-1/LAI and used as effector cells, as in the experiment from figure 8. Different media were used as diluent for cells and T20 during the contact with CD4 negative BeWo cells. The media used were either normal culture medium (M) (RPMI 1640 medium), or in 1) 24 h culture supernatant of HIV-1 LAI infected PBMCs, 2) 24 h culture supernatant of uninfected PBMC or 3) 24 h culture supernatant of CD4 negative cells from PBMCs. The figure shows p24 Ag produced in basolateral chamber at day 8 post contact in these different conditions. The histograms are means (with standard deviations to the means) of values from three different wells of one experiment out of two.

The experiment with purified CD4 positive T cells was then performed in the presence of either culture medium as diluent (like on figure 8), or 24-hours culture supernatants from HIV-1 LAI infected PBMCs, 24 hours culture supernatant of uninfected PBMCs, or of 24 hours supernatant of CD4 negative cells from uninfected PBMCs. Results from these experiments are reported on figure 9. These results confirm that T20 is highly potent in blocking HIV-1/LAI transfer between infected purified CD4 positive T-cells and BeWo cells monolayer. Upon addition of 24 hours culture supernatant of PBMCs (infected or not) or of CD4 negative cells from PBMCs, the enhancement of viral passage by T20 was restored, in a dose dependent manner, since less diluted supernatants contribute to higher increase of fusion. These results indicate that a soluble factor secreted by CD4 negative cells present in the PBMC pool contributes to the enhancement of viral passage across the BeWo monolayer by T20.

## Discussion

The present report shows that CCR5 antagonists and fusion inhibitors block, but with a moderate efficacy, HIV-1 cell-to-cell transmission during the contact between R5 or R5X4 infected PBMCs in contact with a polarized monolayer of BeWo cells. The CXCR4 antagonist evaluated in the present study also potently inhibited HIV-1 transmission from X4-viruses infected PBMCs interacting with BeWo cells. All these drugs were efficient with IC50 in the range of 4 to 20,000 nM. The present study also uncovered an intriguing feature of T20 that we found to increase the transmission of X4 viruses in our *in vitro *model of interaction between BeWo/PBMC-HIV-1X4 that mimics the human trophoblast barrier.

Earlier studies have shown that cell-to-cell HIV infection is more efficient than cell-free virus infection of permissive cells [37]. Thus, our data on increased IC50s of T20, TAK779, SCH-350581 to block R5 and R5X4 HIV-1 cell-to-cell transmission as compared to cell-free PBMC infection is consistent with these observations.

The two CCR5 antagonists failed to inhibit PBMC infection with the dualtropic isolate A204, but were efficient, though at high concentrations, to block cell-to-cell viral transmission of the same isolate. This difference could be explained by the homogeneity of BeWo cells, as compared to the mixture of cell populations with different properties present in PBMCs preparation. It has been recently shown a preferential use of CXCR4 by R5X4 HIV-1 isolates in primary lymphocytes [35]. In the present study, we characterized by a functional assay [38], the coreceptor usage of the clinical isolate A204. Of the 20 clones we analyzed, all were able to use both CCR5 and CXCR4 to infect U87.CD4-CXCR4 or U87.CD4-CCR5 (data not shown).

Another feature of the two CCR5 antagonists is the difference in their distinct efficacies to block cell-free HIV-1 PBMC infection and cell-to-cell transmission of the virus in our system. The IC50 for SCH-350851 necessary to block BeWo/PBMC-HIV-1 Ba-L passage is 18 to 22 folds higher than that needed to inhibit HIV-1 Ba-L/PBMC infection. On the contrary, for TAK779, only 1 to 1.5 times more drugs were needed to achieve 50% inhibition of BeWo/PBMC-HIV-1 Ba-L passage. The difference could be due to particular physico-chemical properties of the molecules, or to a subtle difference in the mode of action of the two molecules. It has been shown that both drugs inhibit infection of PBMCs by R5 viruses by binding to various amino acids residues of the transmembrane domains 1, 2, 3, 5 and 7 of the CCR5 molecule, preventing viral entry [39,40]. Given these latter observations, the difference in activity we observed between the two CCR5 antagonists should lay in their intrinsic physico-chemical properties rather than the targets amino-acids on the CCR5 molecule.

In line with published data obtained with different infection systems, our data here show that the prototype CXCR4 antagonist AMD3100 can also efficiently inhibit the viral passage across the BeWo cell monolayer in contact with PBMCs infected by X4 or R5X4 HIV-1. Given the progressive emergence of X4 viruses in the course of AIDS disease even under HAART [41], the availability of a potent CXCR4 antagonist as part of salvage therapy is of utmost interest. However, AMD3100 does not seem to be the best candidate for this purpose because of its paucity in reducing plasma viral load in a clinical trial [42]. A concern also remains on the effects of AMD3100 on haematopoiesis, as this molecule has been shown to be a powerful and fast mobilizer of CD34+ progenitor cells, beside its potential anti X4-HIV-1 activity [43].

The mechanism by which T20 blocks HIV-1/target cell membrane fusion has been extensively explored [44,45]. The general consensus is that T20 (and C34) are effective once HIV-1 gp120-CD4 has taken place, inducing a conformational change of the heterodimer gp120-gp41 [36]. This conformational change allows, to its turn, the exposition of otherwise masked regions of gp41, especially the N- and C-terminal Heptad repeats (NHR and CHR, respectively) domains [36,46]. This so-called pre-hairpin intermediate is stable enough to be targeted by antibodies and inhibitors [47]. Instead of the normal interaction between N- and C-terminal HRs to yield a six-helix bundle for membrane fusion and viral genetic material transfer into the host cell, T20 (and C34) binds to the NHR, preventing the formation of the six helix bundle and thus membrane fusion. Our present results with HIV-1 Ba-L and HIV-1 A204 are consistent with that model for BeWo-CD4 positive cells, as a dose-dependent inhibition of viral entry was observed, though with IC50s that are 1,000 fold higher than those reported for classical HIV-1/PBMC infection. Half maximal inhibitory concentrations (3–5 μM for CD4 negative target cells) remain, however, in the range of T20 pharmacokinetics values with standard subcutaneous dose of 90 mg twice daily [48]. The fact that T20 was efficient in preventing R5 and R5X4 passage even if the target BeWo cells were CD4 negative suggests that another mechanism of inhibition distinct from the one outlined above might be involved in the context of HIV-1 cell-to-cell transmission. This alternative mechanism of T20 action during HIV-1 R5 and R5X4 infected PBMC and CD4 negative BeWo cells, might imply other receptors like Galactosyl Ceramide, expressed on the surface of these cells [49,50]. Although efficient, though at high concentrations, on R5 and R5X4 viruses, T20 was not effective in blocking viral passage in X4 HIV-1 infected PBMC in interaction with BeWo cells. Rather, T20 (and C34), enhanced it.

This enhancement of X4 HIV-1 passage by T20 and C34 following cell-to-cell contact was unexpected. This enhancement is CD4 independent. In a recent work by Blanco and colleagues [51], a high level of coreceptor-independent HIV transfer was observed between primary CD4+ T-cells. This transfer was enhanced up to 300 folds by C34 but also by the CXCR4 antagonist AMD3100. The enhancement was only observed with the X4 virus NL4-3. Our present observations are different in the way that AMD3100 blocked viral passage across the BeWo cell monolayer in contact with X4 HIV-1 infected PBMCs. We cannot, however, exclude that AMD3100 could also increase X4 HIV-1 passage in our model at higher doses (10 μg/ml) like in the work reported by Blanco and colleagues. Also, entry enhancement we observed is coreceptor dependent and CD4-independent, the exact opposite of transfer enhancement reported by Blanco and colleagues. It has also been shown that X4 virus were more sensitive to inhibition by T20 and C34 in cell-free PBMC infection [52]. This higher sensitivity of X4 virus to fusion inhibitors is thought to lay, at least in part, to the direct interaction of T20, but not C34, with the CXCR4 molecule on the cell surface [52]. This "chemokine" aspect of T20 needs further investigation, complementary to published data [53].

From our results, we cannot exclude a direct interaction of T20 with CXCR4 present on target BeWo cells because the enhancement was only observed with X4 virus and could be inhibited in the presence of AMD3100 (not shown). This entry enhancement was not due to the polarized state of BeWo cells, as we had similar observations with non-polarized cells on slides. Rather, T20 (and C34), could first attract effector cells infected with an X4 HIV-1 towards target cells, followed by an increased of cell-membrane fusion. It has been reported that T20 can act as a chemo attractant of monocytes, but not of lymphocytes [54]. This observation partly justifies our experiment with purified CD4 positive T cells.

Interestingly, when we used these purified CD4 positive T cells (instead of PBMCs) infected with HIV-1/LAI as effector cells in our system, T20 was found to be highly potent in inhibiting viral passage across the BeWo monolayer (Fig. 8). The enhancement of viral passage across the BeWo monolayer by T20 could thus be a result of direct interaction between X4 HIV-1 infected CD4+ T cells, another cell population (CD4 negative) within the PBMCs and the BeWo cells. Alternatively, enhancement of X4 HIV-1 passage by T20 and C34 could be due to the synergistic effect of T20 (and C34) and a soluble factor secreted par CD4 negative cells present in the PBMCs. In our last experiment (9), 24h-cultures supernatants from CD4 negative cells were added during the interaction between CD4 positive T cells infected with HIV-1/LAI and CD4 negative BeWo cells. In these conditions, the enhancing effect of T20 was restored, suggesting that a soluble factor secreted by CD4 negative cells contributes to the increase of X4 HIV-1 passage in the presence of T20. These findings need, however, further investigations.

The precise mechanism by which the two C-terminal Heptad Repeat peptides (T20 and C34) enhanced X4 HIV-1 passage across the BeWo cell monolayer remains to be elucidated. However, this observation is of real concern, if confirm by others, with regards to HIV-1 transmission at the level of epithelial surfaces, during sexual or mother-to-child transmission for example, in patients under Enfuvirtide therapy. Other abnormal peculiarities of HIV-1 in the presence of T20 have been reported. Some HIV-1 strains whose abnormal envelope folding, leading to viruses unable to properly matured, were rescued by T20 addition [55].

In the specific case of HIV-1 PMTCT, the use of Enfuvirtide is very limited to date. Recently however, studies reported the use of T20, on a backbone of combined therapies [56-58]. The study by Cohan and colleagues reported the case of a mother infected with a multidrug resistant HIV-1 and who transmitted the virus to her child, despite plasma viral load suppression by T20 treatment. The phenotype of the transmitted virus was not discussed, but the eight clones randomly selected and sequenced in gp41 HR1 and HR2 were all T20 sensitive. The study by Brennan-Benson and co-workers, reported on two cases of pregnant women infected by multidrug resistant HIV-1, with the delivery of two uninfected children. The plasma viral load was fully suppressed for one case and lowered down to 300 copies/ml for the other. In this study too, the viral phenotypes were not discussed. The other interesting observation from this study is the lack of transplacental passage of T20. Thus, from these studies, it is difficult to conclude on the potency of T20, with a weak if any, transplacental transfer, in preventing HIV-1 mother-to-child transmission.

## Conclusion

Our *in vitro *data showed that HIV-1 co-receptor antagonists (TAK779, SCH-350581 and AMD3100) are active at high doses to block viral passage during cell-to-cell contact across a monolayer of placenta-derived cell line in contact with PBMCs infected with R5, R5X4 and X4 viruses. HIV-1 entry inhibitors are less efficient, *in vitro*, to block cell-to-cell virus transmission than cell-free HIV-1 infection of PBMCs and CCR5 antagonists do not prevent PBMC infection by dual tropic HIV-1 in contrast to cell-to-cell infection in our model. Further steps of evaluating these molecules include the assessment of the compatibility of these high doses of drugs with the placental tissue viability *ex vivo*. The finding that T20 was less potent than expected on R5 and R5X4 viruses and counterproductive on X4 viruses in our model raises the question of its use at mucosal interfaces.

## Materials and methods

### Material

#### Cells

The human choriocarcinoma BeWo cell line (ATCC CCL 98) [59] was obtained initially from the American Type Culture collection (Rockville, MD). The cells were maintained in Dulbecco's modified Eagle medium (GIBCO, Invitrogen SARL, Cergy Pontoise, France) containing glucose (25 mM), supplemented with 20% heat-inactivated foetal calf serum (GIBCO), 20 mM L-Glutamine, 50IU penicillin and 50 μg/ml streptomycin at 37°C, in 5% CO2 humidified atmosphere. A sub-cell line expressing the CD4 molecule on the cell surface was generated by transfection of this primary cell line, with the plasmid containing the human CD4 sequence (PCMV/CD4SrpuroR) as previously described [31]. Human Peripheral Blood Mononuclear Cells (PBMCs) were prepared from venous blood of healthy donors (Etablissement Français du Sang, Rungis, France. Habilitation #HS2003-5240) on Ficoll gradient (PAA laboratories, Les Mureaux, France) and used either to produce virus stocks, or as effector or as indicator cells. PHA-stimulated PBMCs were cultivated in RPMI1640 medium supplemented with 10% heat-inactivated foetal calf serum and 100 IU/ml of recombinant human IL-2 (Chiron B.V. Amsterdam, The Netherlands).

#### Viruses

The viral isolate HIV-1 Ba-L (R5), a primary isolate of HIV-1 LAI (X4), the clinical isolate HIV-1 A204 (R5X4) from a mother who transmitted *in utero *the virus to her child [60] and HIV-1 KH025 (X4) from a Cambodgian patient [61], were used throughout the work.

#### Entry inhibitors

The following reagents were obtained through the NIH AIDS Research and Reference Reagent Program, Division of AIDS, NIAID, NIH: AMD3100, CXCR4 antagonist, reagent #8128; TAK779, CCR5 antagonist, reagent#4983, T20, fusion inhibitor, reagent#9845 and C34, fusion inhibitor, reagent#9824. The CCR5 antagonist SCH-350581 [40] was kindly provided to us by Schering-Plough Research Institute (Kenilworth, NJ).

### Methods

#### Cell cultures

Human PBMCs, BeWo cell lines (either expressing at their surface the CD4 molecule or not) were cultured as described previously [4]. The level of CD4 expression on BeWo cells was systematically controlled by flow cytometry before seeding on Transwell^® ^inserts by using an anti-CD4 antibody labelled with PC5 dye (Beckman-Coulter, Villepinte, France). The level of surface CD4 expression on BeWo-CD4+ cells was always between 15 and 40%. The U87 cell line transfected to stably express CD4 (U87-CD4) [31] on cell surface was used as positive control.

#### Purification of CD4+ T cells

CD4 positive T cells were purified from a healthy donor's PBMCs, by positive selection with an anti-CD4 labelled magnetic beads (Miltenyi Biotec Inc. Auburn, CA, USA) followed by negative selection with an anti-CD14 labelled magnetic beads (Dynal Biotech ASA. Oslo, Norway). Cells obtained were > 98% CD3+/CD4+ T-cells as confirmed by flow cytometry.

#### Inhibition by different ARVs of PBMC infection with cell-free HIV-1

To verify biological activities of the different coreceptor antagonists in a known system, 10^5 ^PHA stimulated PBMCs were pre-incubated for 30 min in the presence of variable doses of the different entry inhibitors, then infected with the different viral isolates at multiplicity of infection (MOI) of 10^-3 ^and 5 × 10^-3 ^for one hour. For the inhibition with fusion inhibitors, the different viral isolates, at 10^-3 ^and 5 × 10^-3 ^MOI, were first pre-incubated with the drugs for 30 mn before the contact with PBMCs as for inhibition with coreceptor antagonists. Cells were then washed 3 times with RPMI1640 (GIBCO) and plated in the presence of the same concentration of entry inhibitor they were pre-incubated with. After 3 days at 37°C, supernatants were collected and replaced with fresh medium containing IL-2, without drug, until day 7. Cultures were then stopped and viral production checked by p24 antigen ELISA (Beckman-Coulter) quantification in the supernatants.

#### Inhibition of cell-to-cell infection by entry inhibitors

CD4+ and CD4- BeWo cells (target cells) were seeded at 2.5 × 10^5 ^and 5 × 10^5 ^per cm^2^, respectively, in the inserts of a 12-well Transwell^® ^system (Corning SA, Avon, France), five days before the contact with infected PMBC (effector cells). Preliminary experiments have indeed shown that the two cell lines of BeWo present different growth kinetics. The day of contact, BeWo cells on the Transwell^® ^insert (apical and basolateral chambers) were equilibrated in RPMI1640 medium containing IL-2 for 1–2 h, then pre-incubated for 30 min with variable doses of coreceptor inhibitors. Then, HIV-1 (Ba-L, LAI, A204 or KH025) infected PBMCs (day 8 post infection), were equilibrated in the drug solution at appropriate concentration, and added at the apical chamber of the Transwell^® ^system and incubated 3 h at 37°C. For inhibition with fusion inhibitors, effector cells were pre-incubated with the drugs (37°C, 30 mn) before the contact with target cells. For CD4+ and CD4- BeWo cells, 5 × 10^5 ^and 10^6 ^infected PBMCs were added, respectively. Thus, the effector-to-target ratio of 2 remains the same for the two cell lines. At the end of the 3 h contact period, the inserts were transferred onto new 12-well plates containing fresh RPMI1640 medium without IL-2 and extensive washings (up to seven, including a 1 minute trypsin treatment) were performed. After the last washing, inserts were transferred onto wells containing 1.5 × 10^6 ^PHA stimulated HIV negative PBMCs as indicator cells in culture medium with IL-2. No inhibitor was added. In experiments with purified CD4+ T-cells, 24 h cultures supernatants of uninfected PBMCs, or LAI-infected PBMCs or uninfected CD4+ T-cell depleted PBMCs, were used as diluents for T20 and cells during the contact period. Inserts were left on indicator cells for 5 days, with half of apical and basolateral media changed by fresh RPMI1640+IL-2 medium at day 2 post-contact. At day 5, the inserts were removed, controlled for the integrity of BeWo cells monolayer visually and by the Evans blue passage test, as previously described [4]. Half the volume of supernatants from the basolateral chambers were harvested and replaced by fresh RPMI1640+IL-2 medium. This was repeated at day 8 and 12 post-contact and viral production in the supernatants was checked by p24 antigen quantification.

#### Fluorescent dye transfer experiments

These experiments were performed on 8-well LABTEK slides (Nalge Nunc International, Rochester, NY). Slides were coated overnight at 4°C with 10% bovine collagen in PBS (Sigma Aldrich Chemical Co., L'Isle d'Abeau Chesnes, France) and then washed 3 times with PBS, air-dried, and kept at room temperature until use.

2 × 10^4 ^BeWo cells (CD4- or CD4+) in 200 μl of medium were seeded per well for 24 h at 37°C, 5% CO2. The protocol used was adapted from the one by Weiss et al [62]. Human PBMCs, at day-7 post infection with HIV-1 LAI, were labelled with the green cytoplasmic dye calcein-AM (Sigma-Aldrich) at 1.5 μg/ml in PBS. After 30 minutes of labelling, cells were washed extensively with PBS and used as effector cells. Two hours before the contact between effector cells (HIV-1 infected PBMCs) and target cells (BeWo cells), the latter were equilibrated in RPMI1640 medium containing IL-2. The contact was performed at an effector-to-target ratio of 3 (a titration was performed in the range of 1–5 E:T ratios) in the presence or not of drugs. Cells were left in contact for 4 h at 37°C, 5% CO2. After the 4 hours, cells were fixed with 2% (v/v) Paraformaldehyde in PBS for 30 mn at room temperature, washed 3 times with PBS, air-dried and counterstained with the nuclear dye 4'-6'-diamidino-2-phenylindole, dihydrochloride (DAPI), FluoroPure™ grade (Molecular Probes, Eugene, OR) at a final concentration of 300 nM in PBS for 5 min. The slides were then washed with PBS and mounted in the presence of the anti-fade ProLong^® ^Gold Antifade Reagent (Molecular Probes, Eugene, OR) and left to cure overnight at 4°C in the dark before viewing. Slides were viewed on a Nikon FXA fluorescent microscope at 20× (objective) and 10× (ocular). The microscope was coupled to a Nikon D1 numeric Camera. Images were captured on an Apple^® ^PowerMacintosh^® ^computer by using the Nikon Capture^® ^and Adobe Photoshop^® ^softwares. Green fluorescence was quantified in light units (pixels) inside (fusion) and outside (adhesion) BeWo cells from 3 different fields for each experimental conditions: in the absence of drug, in the presence of 10 μM T20 or 800 nM AMD3100.

## Competing interests

The author(s) declare that they have no competing interests.

## Authors' contributions

AA designed and performed experiments, drafted and reviewed the manuscript. CC and MTN performed experiments. FBS designed experiments and reviewed the manuscript. EM designed and performed experiments, drafted and reviewed the manuscript. All authors read and approved the final manuscript.
